# Sex-Dimorphic Kidney-Brain Connectivity Map of Mice

**DOI:** 10.1007/s12264-024-01240-z

**Published:** 2024-06-19

**Authors:** Xulin Li, Yuan Zhou, Feng Wang, Liping Wang

**Affiliations:** 1 Shenzhen Key Laboratory of Neuropsychiatric Modulation, Shenzhen-Hong Kong Institute of Brain Science, Shenzhen Institute of Advanced Technology, Chinese Academy of Sciences, Shenzhen, 518055 China; 2 CAS Key Laboratory of Brain Connectome and Manipulation, the Brain Cognition and Brain Disease Institute, Shenzhen Institute of Advanced Technology, Chinese Academy of Sciences, Shenzhen, 518055 China; 3 Guangdong Provincial Key Laboratory of Brain Connectome and Behavior, the Brain Cognition and Brain Disease Institute, Shenzhen Institute of Advanced Technology, Chinese Academy of Sciences, Shenzhen, 518055 China; 4 University of Chinese Academy of Sciences, Beijing, 101408 China

**Keywords:** Kidney innervation, Sexual dimorphism, Pseudorabies virus, Central neural system

## Abstract

The kidneys are essential organs that help maintain homeostasis, and their function is regulated by the neural system. Despite the anatomical multi-synaptic connection between the central autonomic nuclei and the kidneys, it remains unclear whether there are any variations in neural connections between the nervous systems and the renal cortex and medulla in male and female mice. Here, we used the pseudorabies virus to map the central innervation network of the renal cortex and medulla in both sexes. The data revealed that specific brain regions displayed either a contralateral-bias or ipsilateral-bias pattern while kidney-innervating neurons distributed symmetrically in the midbrain and hindbrain. Sex differences were observed in the distribution of neurons connected to the left kidney, as well as those connected to the renal cortex and medulla. Our findings provide a comprehensive understanding of the brain-kidney network in both males and females and may help shed light on gender differences in kidney function and disease susceptibility in humans.

**Supplementary Information:**

The online version contains supplementary material available at 10.1007/s12264-024-01240-z.

## Introduction

Homeostasis is a crucial aspect of the body’s survival and various physiological processes. It is a complex system that involves intricate feedback circuits, regulatory systems, and adaptive responses that work together to ensure the body’s vital parameters remain within a narrow range. The kidneys play a vital role in maintaining homeostasis through their regulation of fluid and electrolyte balance, acid-base balance, blood pressure regulation, and erythropoiesis regulation. Abnormality in renal functions led by multiple primary or secondary injuries to the kidneys perturbs homeostasis and results in life-threatening diseases.

The kidney’s functions are regulated in three major ways: autoregulation [1], hormonal, and neural system. Neural regulation is particularly important in kidney homeostasis as it regulates tubular sodium reabsorption, renin secretion, and renal blood flow, all crucial components in maintaining homeostasis. Recent studies have shown that sympathetic renal nerve activity increases under pathological conditions, including renal [2], cardiovascular, and metabolic diseases [3–6]. Moreover, renal denervation has been proposed as an adjunct treatment option in uncontrolled resistant hypertension by the European Society of Cardiology recently [7]. However, the precise characteristics of the central network innervating the kidneys still need to be depicted.

The brain controls the central network innervating the kidneys *via* inhibitory and excitatory reno-renal reflexes that contribute to maintaining normal renal functions. Several brain and spinal cord regions innervating the kidney have been identified using dyes [8], tracer proteins [9], or neurotropic viral tools [8,10]. The studies collectively depicted a central autonomic network related to the kidney, including the ventromedial medulla (VMM), rostral ventrolateral medulla (RVLM), A5 cell group, and the hypothalamic paraventricular nucleus (PVN) in the brain, as well as the intermediolateral cell column (IML), the lateral funiculus (LF) and the intercalated cell column (IC) in the spinal cord.

Although studies have been performed on rats to investigate renal functions, most of them were conducted on males. Sex differences in renal functions have been recognized since the 1940s, but the exact mechanism remains to be elucidated. Evidence suggested that sex differences occur under normal circumstances, such as renal sodium handling in different segments of the renal tubule and sensitivity to arginine vasopressin [11,12], as well as pathological states, such as more severe progression of renal ischemia-reperfusion injury in males [13,14]. Additionally, previous neural tracers were not specifically restricted to the renal cortex or medulla, and they were injected dispersedly. However, the vasculature and tubular system exhibit significant heterogeneity between the cortex and the medulla.

To address these limitations, Jia *et al.* developed pseudorabies virus (PRV)-531 and PRV-724 which are more stable and have better labeling efficiency than previous PRV-152 and PRV-614 [15]. In the present work, these strains were used to identify kidney-innervating neurons in both male and female mice, and differences in central nervous system (CNS) neural network innervating the renal cortex and medulla were compared. We identified 34 brain regions that were labeled, providing a comprehensive atlas of kidney-related CNS neural networks that will facilitate therapies for disorders combining renal malfunction and neuropsychiatric symptoms. Our work provided a more accurate and comprehensive atlas of kidney-related CNS neural networks that will aid in developing targeted therapies for disorders combining renal malfunction and neuropsychiatric symptoms.

## Materials and Methods

### Animals

Virgin male and female C57BL/6J mice, aged 14–18 weeks and consisting of five individuals in each group, were purchased from Zhejiang Vital River Laboratory Animal Technology Co., Ltd (Pinghu, China). The mice were housed in a 12-h light-dark cycle and provided with *ad libitum* food and water. All procedures were performed under protocols approved by the Animal Care Committee of Shenzhen Institute of Advanced Technology (SIAT), Chinese Academy of Sciences (IACUC number: SIAT-IACUC-20221010-NS-NTPZX-WLP-A2137-02).

### Viral Injections

PRV recombinants, PRV-531 (with EGFP fluorescent tag) and PRV-724 (with mRuby fluorescent tag) were purchased from BrainCase Co., Ltd (Wuhan, China). Animals were weighed and anesthetized with 1% pentobarbital and placed on a 37 °C-heat pad. Hair was shaved and a 1 cm incision was made on the back to expose the kidney. Sterile cotton soaped with sterile normal saline was used to keep the tissue moist. A total volume of 1 µL of each virus strain (10^10^ plaque-forming units/mL) [16] was injected into a single site of the kidney. For comparison of innervation of left and right kidneys, PRV-531 was injected into the cortex (1 mm below the surface of the kidney) of the upper pole of the left kidney, and PRV-724 into the corresponding site of the right kidney. For comparison of innervation of the cortex and medulla, PRV-531 was injected into the cortex and PRV-724 was injected into the medulla (2 mm below the surface of the kidney). To prevent leakage of the virus, the contact surface of the microinjection needle and the kidney was sealed with Kwik-Sil (WPI, Sarasota, US), and a needle was held in place for an extra 5–10 min before it was slowly pulled out. After the removal of the needle, the injection site was swabbed with sterile cotton to prevent any leakage. Then the kidney was placed back into the abdomen, and the incision was sealed.

### Tissue Preparation

The animals were anesthetized with 1% pentobarbital and then transcardially perfused with PBS and then paraformaldehyde (PFA, 4% weight/volume, Boster, Wuhan, China). The brains were harvested and post-fixed with 4% PFA overnight. After cryoprotection with 30% sucrose (weight/volume in PBS), the brains were sectioned into four series of 30 µm slices. The slices were stained with DAPI and mounted for imaging.

### Microscopic Imaging and Neuron Quantification

The prepared slices were imaged with an automated slide scanner (Olympus, Tokyo, Japan) under 10× objective lens amplification. All the labeled neurons were manually counted by the experimenter blind to the experiment design.

### Statistics

Data were analyzed using GraphPad Prism 8.3.0 (RRID: SCR_002798) (Boston, US) with the two-way analysis of variance (ANOVA) followed by the Holm-Sidak *post hoc* multiple comparisons test. ****P <0.0001, ***P <0.001, **P <0.01, and *P <0.05 were considered as statistically significance in all tests.

### Data Availability

The data supporting this study’s findings are available from the corresponding author upon reasonable request.

## Results

In the current study, a survival rate of >90% for animals 5 days after PRV injection was found. The whole brain was screened, and 34 brain regions were identified as being infected by PRV (Table 1). The count of infected neurons was manually quantified in an unbiased manner. The distribution of kidney-related neurons was concentrated in the pons, medulla, and hypothalamus, consistent with previous reports by other research groups[8–10]. Interestingly, no significant differences were observed in the number of infected neurons between male and female mice in these major divisions (Fig. 5). These results indicated that renal function is a fundamental and conservative component of life.

**Table 1:** Abbreviations for all brain regions mentioned in the article

M1	Primary motor cortex
S1	Primary somatosensory cortex
OVLT	Organum vasculosum laminae terminals
BNST	Bed nucleus of the stria terminals
CeA	Central nucleus of the amygdala
MPA	Medial preoptic area
LPO	Lateral preoptic area
PVN	Paraventricular nucleus
LH	Lateral hypothalamic area
PSTh	Parasubthalamic nucleus
DM	Dorsomedial hypothalamic
PH	Posterior hypothalamic nucleus
RMC	Red nucleus, magnocellular part
EW	Edinger-Westphal nucleus
dmPAG	Dorsomedial periaqueductal gray
lPAG	Lateral periaqueductal gray
vlPAG	Ventrolateral periaqueductal gray
PnO	Oral pontine reticular nucleus
SubCD	Dorsal subcoeruleus nucleus
SubCV	Ventral subcoeruleus nucleus
LDTgV	Laterodorsal tegmental nucleus, ventral part
ROb	Raphe obscurus nucleus
RMg	Raphe magnus nucleus
RPA	Raphe pallidus nucleus
LC	Locus coeruleus
Bar	Barrington’s nucleus
MVePC	Medial vestibular nucleus, parvicellular part
MVeMC	Medial vestibular nucleus, magnocellular part
IRt	Intermediate reticular nucleus
Gi	Gigantocellular reticular nucleus
LRt	Lateral reticular nucleus
RVLM	Rostral ventrolateral medulla
NTS	Solitary nucleus
DMV	Dorsal motor nucleus of the vagus

### Characteristics of Kidney-related Neuron Distribution Between the Left and Right Kidneys and Their Sexual Dimorphism

**Fig. 1:**
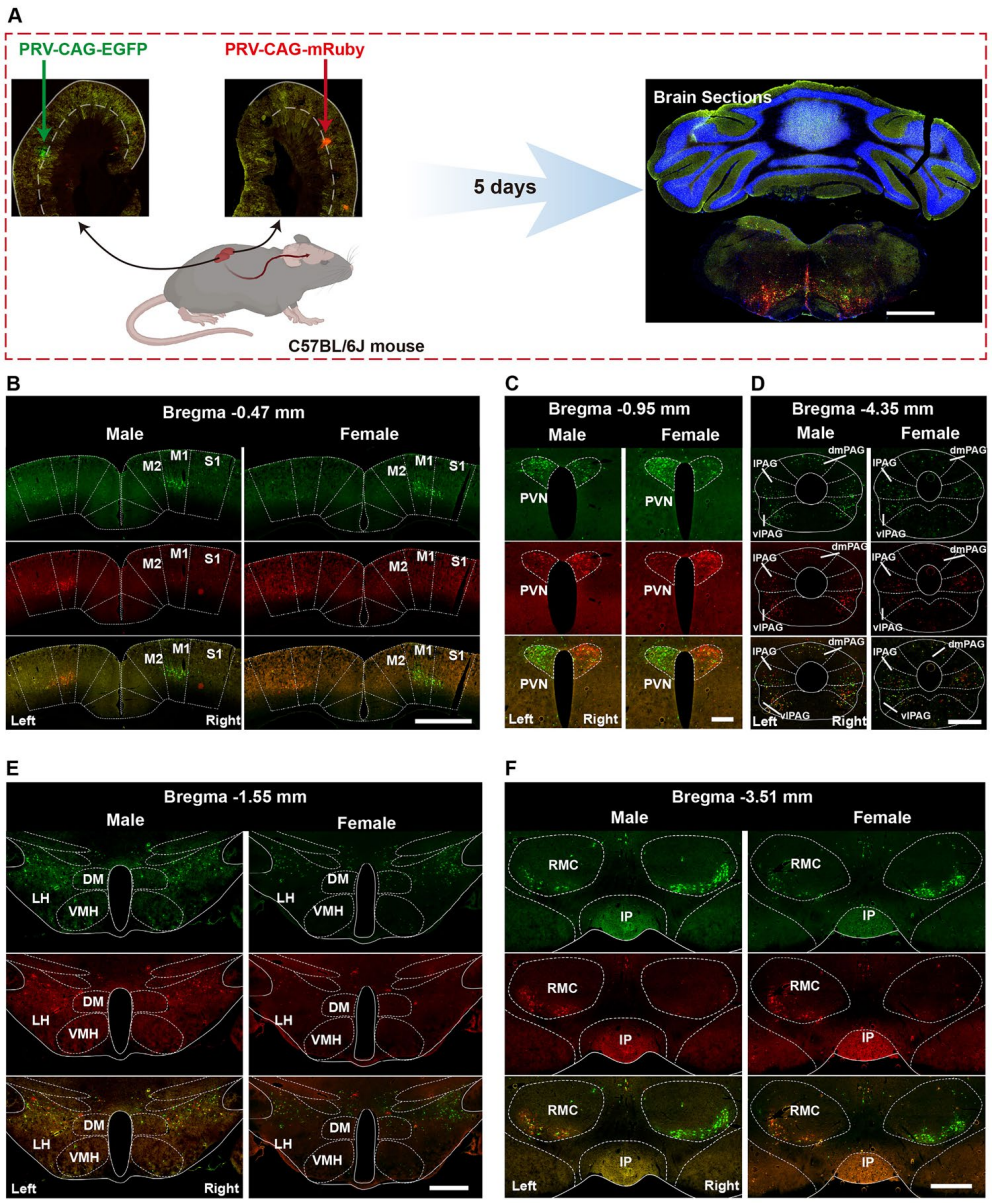
Representative images for the distribution characteristics of kidney-innervating neurons in the motor cortices, PVN, PAG, hypothalamus, and RMC in mouse brains of both sexes. **A** Illustration of the virus injection point at the superior pole of the left or right kidney, and the sample processing schematic. **B, F** Representative images for brain regions of the denser PRV labeling neurons with a dominant contralateral hemisphere PRV labeling. **C** Representative images for brain regions of the denser PRV labeling neurons with a dominant ipsilateral hemisphere. **D, E** Representative images for the brain regions where PRV labeling neurons are distributed symmetrically between the left and right hemispheres. Scale bars, **A** = 1mm, **B** = 250 μm, others are 500 μm.

**Fig. 2:**
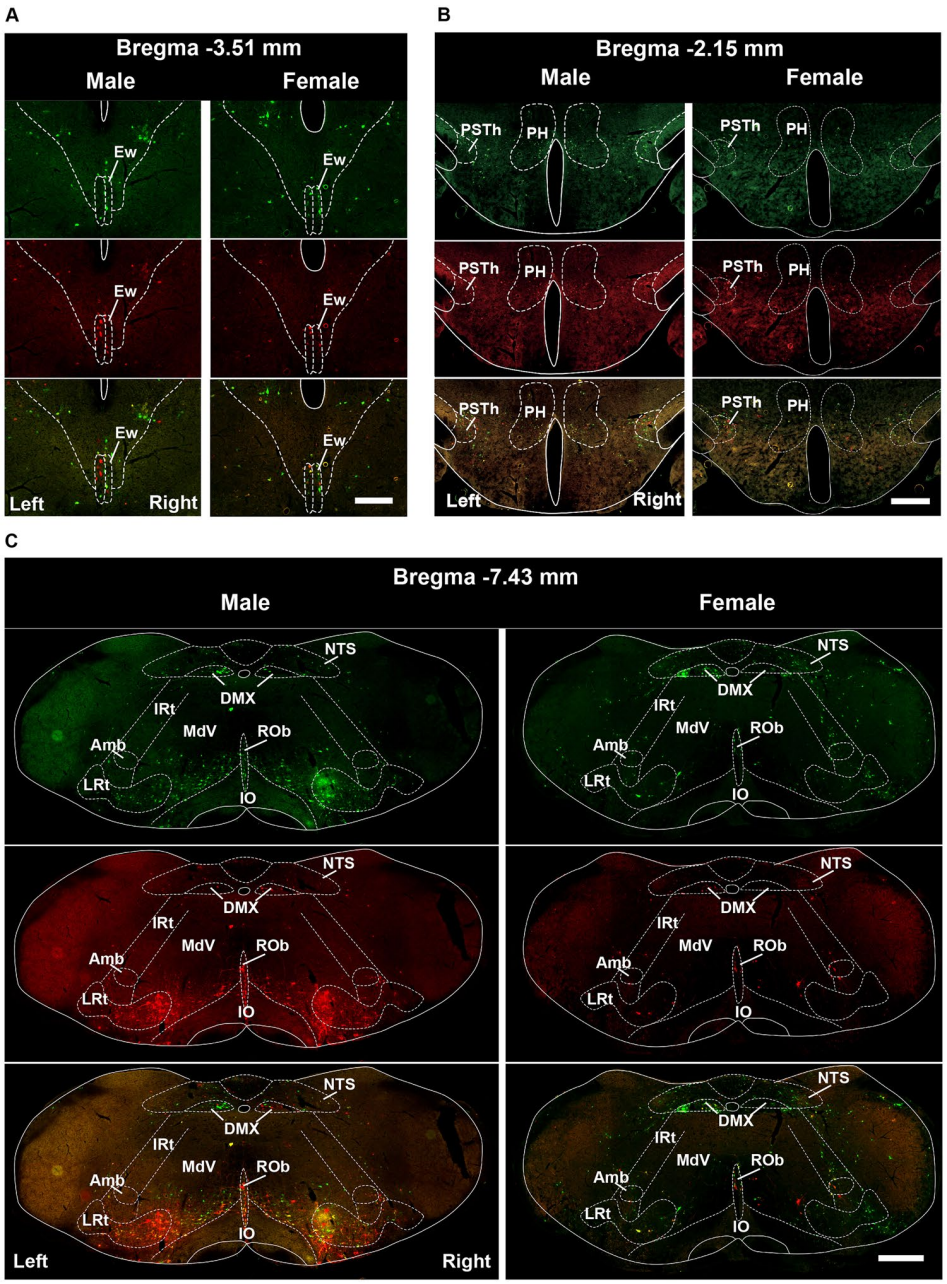
Representative images for the distribution characteristics of kidney-innervating neurons in the hindbrain of male and female brains. **A, B** Images showing PRV labeling neurons in the Ew and the PSTh that are seldom reported in kidney-innervating structures. **C** Images showing the typical regions that are mostly reported in kidney innervation. Scale bars, **A** = 250 μm, others are 500 μm.

To investigate whether there were differences in the way the brain connects to the left and right kidneys, PRV531 (EGFP reporter) was injected into the left kidney and PRV724 (mRuby reporter) into the right kidney simultaneously in male and female mice. After 5 days, the mice were perfused, and fluorescent neurons in the brain were quantified (Fig. 1A). Green fluorescence represented neurons retrogradely labeled from the left kidney, while red fluorescence represented neurons retrogradely labeled from the right kidney. Atlases were created to show general distribution patterns of neurons connected to the left or right kidney in the brain (Fig. S1). Four typical brain regions with asymmetrical neuronal distribution connecting the left and right kidneys were found in all 34 brain regions: the primary motor cortex/primary somatosensory cortex (M1/S1) and the ventrolateral part of the red nucleus, magnocellular part (RMC), the PVN, the parasubthalamic nucleus (PSTh), and the dorsal motor nucleus of the vagus (DMV). The former two nuclei displayed dominant contralateral distribution, while the latter three displayed dominant ipsilateral distribution. We also found virus-labeled neurons located in layer V of the M1/S1 and mainly the magnocellular part of the PVN (Fig. 1B, C). Interestingly, the Edinger-Westphal nucleus (Ew) nucleus and the PSTh nucleus were labeled by the PRV virus of both colors, and the PSTh was densely labeled by the virus retrograded from the ipsilateral kidney. These two nuclei, related to sympathetic regulation, have seldom been mentioned or were omitted in the previous functional studies. Additionally, PRV-labeled neurons were found in either the medial or lateral DMV, known as the preganglionic neural cluster of the vagus nerve, indicating the possibility of vagal innervation to the kidney (Fig. 2).

**Fig. 3:**
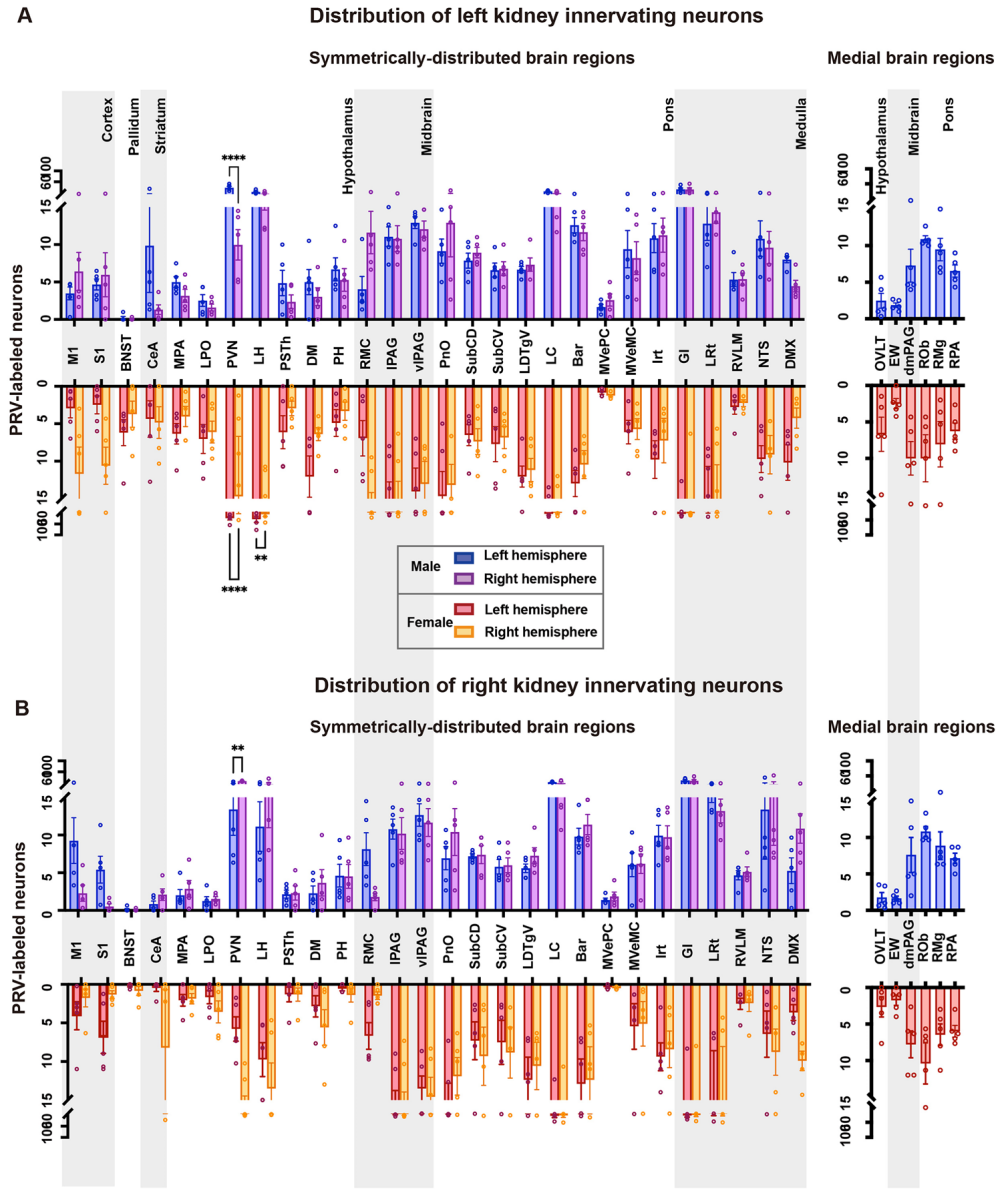
Global inputs to the left and right kidney in both hemispheres of the brain. **A** Quantification of ipsilateral and contralateral EGFP^+^ neurons in the whole brains of both male and female mice. **B** Quantification of ipsilateral and contralateral mRuby^+^ neurons in the whole brains of both male and female mice. Data are shown as the mean ± SEM. *n* = 5 in each group. Two-way ANOVA with Šídák’s *post hoc* test, **P <0.01, ****P <0.0001.

To investigate the distribution of left and right kidney-related neurons in both hemispheres, statistical analyses were performed separately for male and female mice. The results showed that left kidney-related neuron numbers in both the PVN showed statistical differences between the two hemispheres in both sexes, while only the lateral hypothalamic area (LH) showed a statistical difference in female mice (Fig. 3A). However, in the analysis of connections between the right kidney and the two hemispheres, only the PVN in male mice showed a difference between the two hemispheres (Fig. 3B). We also presented the absolute numbers of neurons in the brain connected to the left and right kidneys in male and female mice (Table S1).

**Fig. 4:**
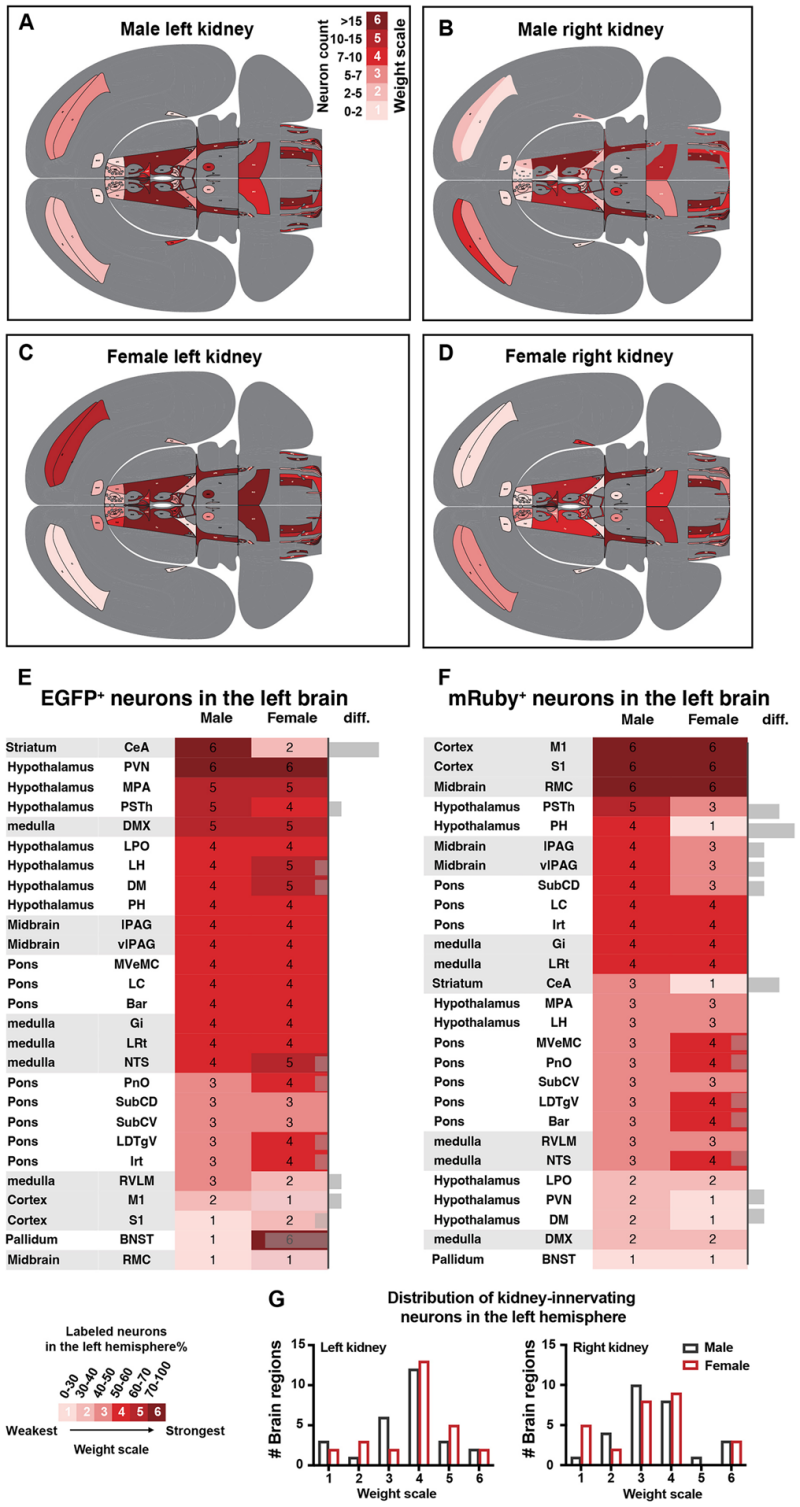
Comparison of left or right kidney-innervating neuron distribution in the left hemisphere. **A**–**D** Representative infected neuron numbers from the left or right kidney in each brain region were divided into 6 bins that indicated the connection weight between the specific region and the kidney. **E**, **F** Data were obtained by calculating the percentage of left brain EGFP^+^ or mRuby^+^ neurons in total neurons in the specific region, which indicates the left or right bias of kidney innervation. The percentage was represented as a 6-pointed scale colored by pink-red connection weight. Grey bars (right columns) indicate left-bias differences between male and female mice. Heatmap showing the percentage of EGFP^+^ or mRuby^+^ neurons in the male and female left brain and difference value between both sexes. **G** Numbers of brain regions falling into different connection weights and their sex differences.

To investigate sex differences in the connecting patterns between the brain to both kidneys, we classified the connection weight into six levels according to the representative infected neuron number in the left hemisphere: scale 1 was for 0–2 neurons, scale 2 for 2–5, scale 3 for 5–7, scale 4 for 7–10, scale 5 for 10–15, and scale 6 for >15 neurons (Fig. 4A–D). The connection weights were then displayed on a mouse brain flatmap created by Hahn [17]. To quantitatively characterize the sex differences in left and right kidney innervation bias, we analyzed the ratio of EGFP^+^ neurons (retrogradely traced from the left kidney) or mRuby^+^ neurons (retrogradely traced from the right kidney) among all brain regions connecting to the left and right kidneys in male and female mice.

The strength of the connections was classified into different intervals based on the ratio of infected neuron number in the left hemisphere *versus* the total infected number in both hemispheres. A 6-point red-pink color scale was used to represent the weight of left or right kidney bias (Fig. 4E–G). We found that there were some differences between males and females in the way certain areas of the brain connected to the kidneys. Specifically, the central nucleus of the amygdala (CeA) and PSTh showed stronger connections to both kidneys in males than in females. In contrast, the oral pontine reticular nucleus (PnO), laterodorsal tegmental nucleus ventral part (LDTgV), and solitary nucleus (NTS) regions showed stronger connections to both kidneys in females than in males. The dorsomedial hypothalamic (DM) region showed a reversed trend, with stronger connections to the left kidney in females and stronger connections to the right kidney in males. For the left kidney, the RVLM and M1 showed stronger connections in males than in females, while the intermediate reticular nucleus (IRt), S1, and bed nucleus of the stria terminalis (BNST) showed stronger connections in females than in males. For the right kidney, the posterior hypothalamic nucleus (PH), lateral periaqueductal gray (lPAG), ventrolateral periaqueductal gray (vlPAG), PVN, and dorsal subcoeruleus nucleus (SubCD) showed stronger connections in males than in females, while the medial vestibular nucleus, parvicellular part (MVePC) and Barrington’s nucleus (Bar) showed stronger connections in females than in males (Fig. 4E–H). Interestingly, most of the nuclei simultaneously connected with both kidneys on a fifty-fifty basis (Fig. 4G), indicating two possible hypotheses that require further verification: either these brain regions regulate functions of both kidneys that compensate for each other, or they act as an information intersection for both kidneys.

**Fig. 5:**
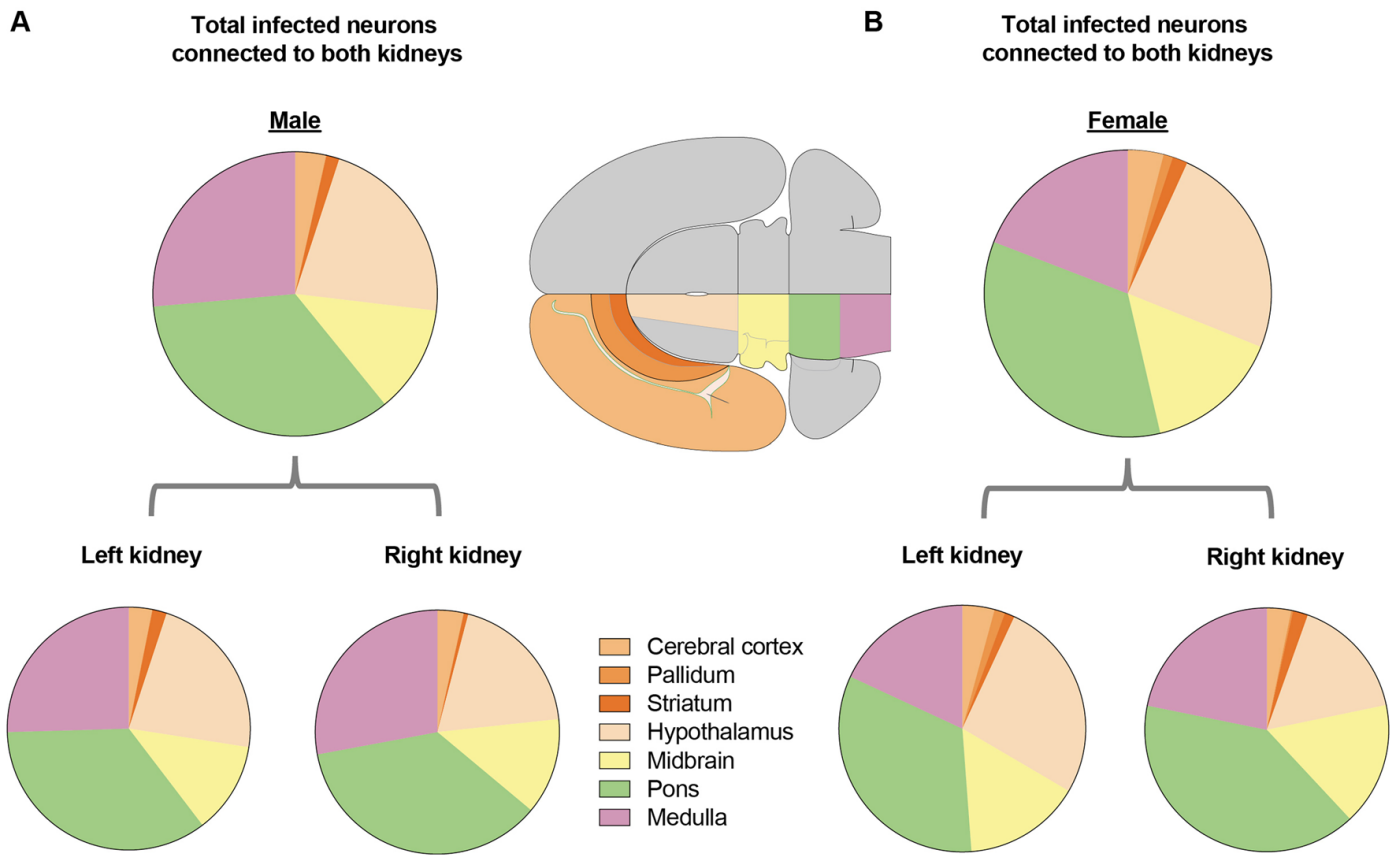
Total innervation intensity in major brain divisions of male and female mice. Pie charts showing 7 major brain divisions containing PRV-infected neurons and the relative proportion of total innervation to both kidneys in male and female mice.

We conducted a further investigation to determine the distribution of kidney-related neurons across seven major brain divisions on a larger scale. The results showed that kidney-related neurons were primarily located in the pons (34.46% for males, 34.50% for females), hypothalamus (21.85% for males, 24.27% for females), and medulla (26.41% for males, 19.14% for females). Interestingly, these patterns were similar in both male and female mouse brains (Fig. 5). These results are consistent with the consensus that the brain controls innate physiological processes and maintains homeostasis of the body by innervating visceral organs in the absence of higher brain functions like consciousness.

### Characteristics of Kidney-related Neuron Distribution Between the Renal Cortex and Medulla and Their Sexual Dimorphism

**Fig. 6:**
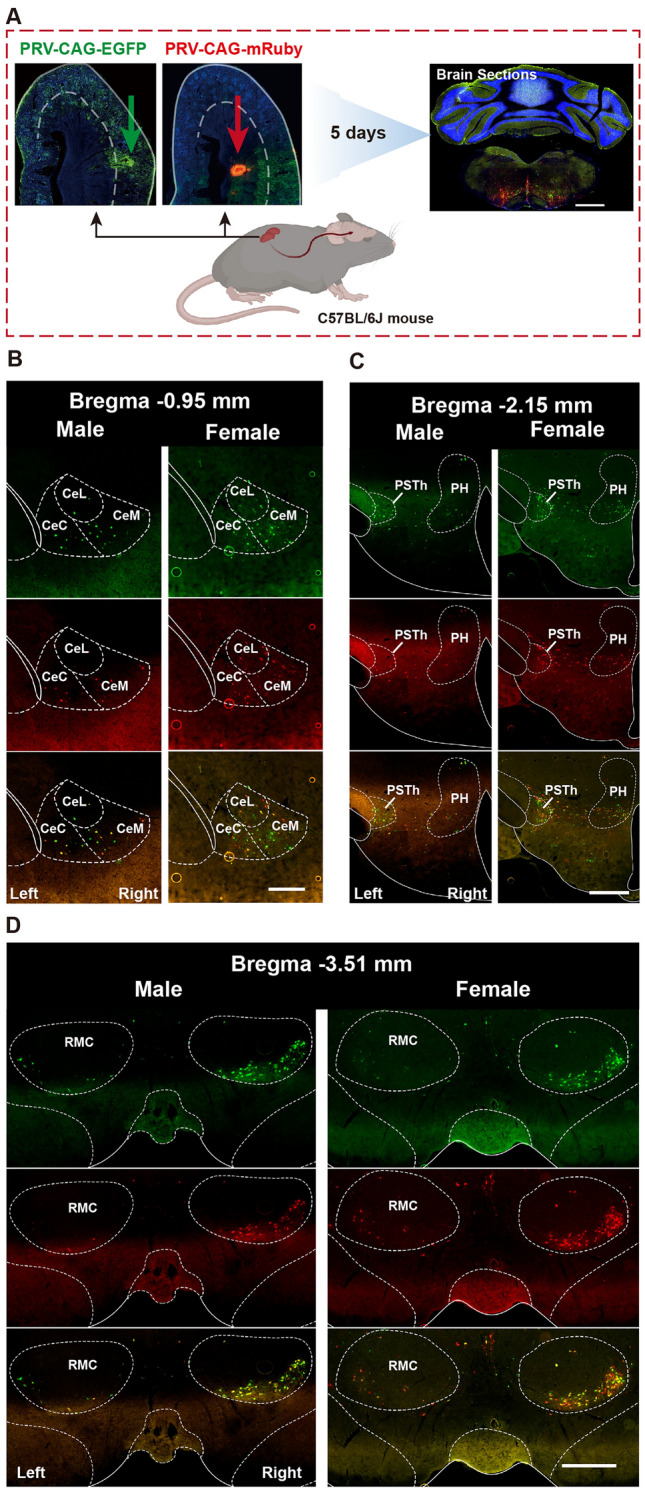
Representative images of the distribution of kidney cortex or medulla-innervating neurons in the male and female brains. **A** Illustration of the virus injection point at the superior pole of the left or right kidney, and the sample processing schematic. **B** Representative images of the distribution of cortex and medulla-innervating neurons in the male and female CeA. **C** Representative images of the distribution of cortex and medulla-innervating neurons in the male and female PH and PSTh. **D** Representative images of the distribution of cortex and medulla-innervating neurons in the male and female RMC. Scale bars, **A** = 1mm, **B** = 250 μm, others are 500 μm.

**Fig. 7:**
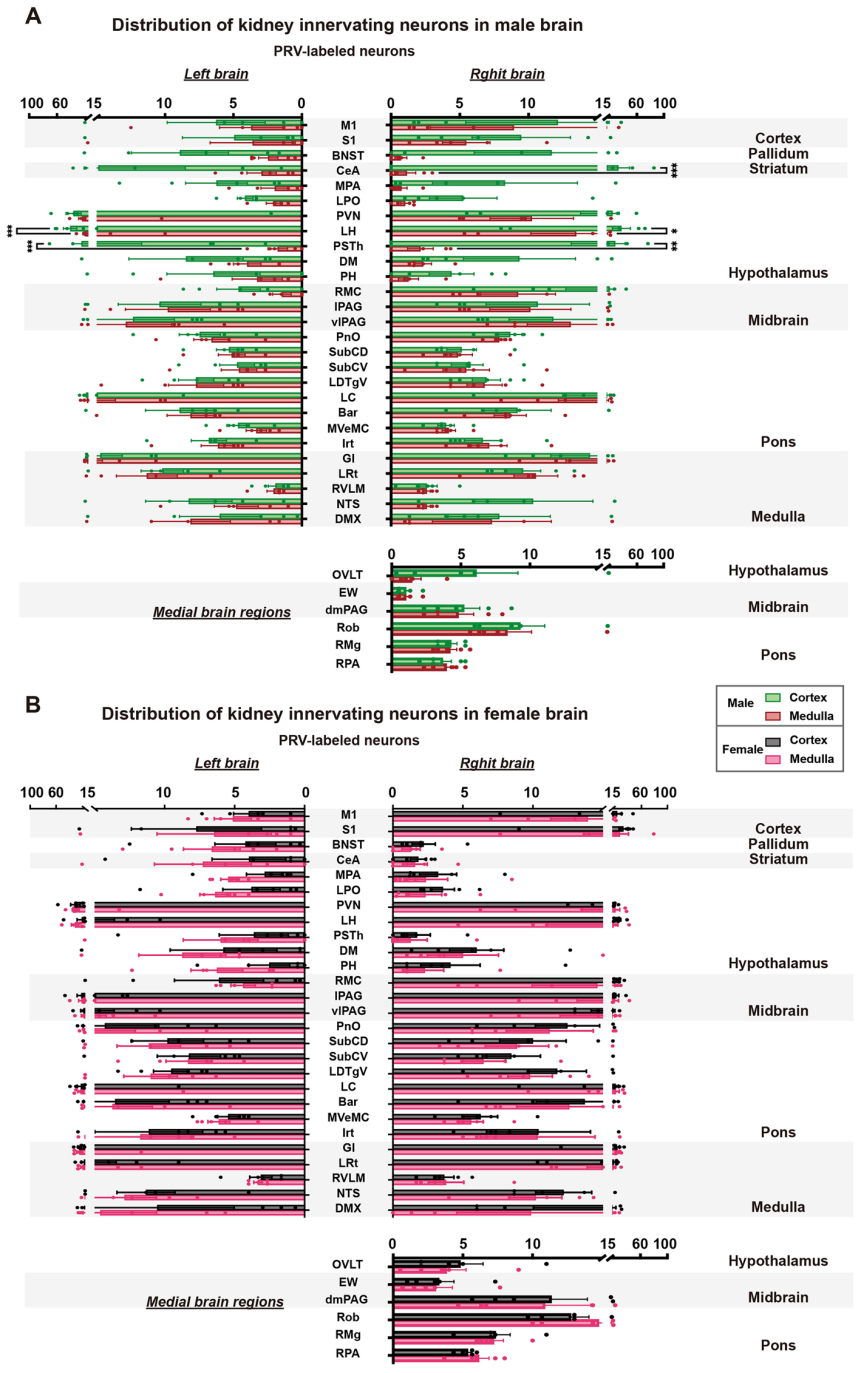
Distribution of neurons that innervate the kidney cortex and medulla. **A** Comparison of the distribution of kidney cortex-innervating and medulla-innervating neurons and its differences between both of the male brain hemispheres. **B** Comparison of the distribution of kidney cortex-innervating and medulla-innervating neurons and its differences between both of the female brain hemispheres. Data are shown as the mean ± SEM. Two-way ANOVA with Šídák’s *post hoc* test. *P <0.05, **P <0.01, ***P <0.001.

To determine whether there were differences in the way the brain connects to the renal cortex and the medulla, which have different structures and functions, PRV531 was injected into the left renal cortex and PRV724 was injected into the left renal medulla of both male and female mice. After 5 days, the mice were perfused and fluorescent neurons in the brain were quantified (Fig. 6A). Green fluorescence represented neurons retrogradely labeled from the left renal cortex, while red fluorescence represented neurons retrogradely labeled from the left renal medulla. We then examined the absolute number of neurons in the brain that are connected to the renal cortex and medulla in both male and female mice, which are presented in Table S2. Finally, the connection between the renal cortex/medulla and brain hemispheres was analyzed in male and female mice. Results showed that neurons related to both the cortex and medulla of the kidney were located in the same brain regions, with similar distribution patterns between the left and right hemispheres (Fig. 6). In the midbrain and hindbrain, neurons that connected with the renal cortex and medulla tended to be the same. However, in the male forebrain, LH and PSTh showed more neurons connected to the renal cortex than to the medulla. The CeA showed more neurons connected to the cortex than the medulla in the right hemisphere of male mice (Fig. 7A). Interestingly, no brain region showed connectivity differences to the renal cortex and medulla in female mice (Fig. 7B).

### Distribution Patterns of Co-labeled Neurons Innervating Both Kidneys

**Fig. 8:**
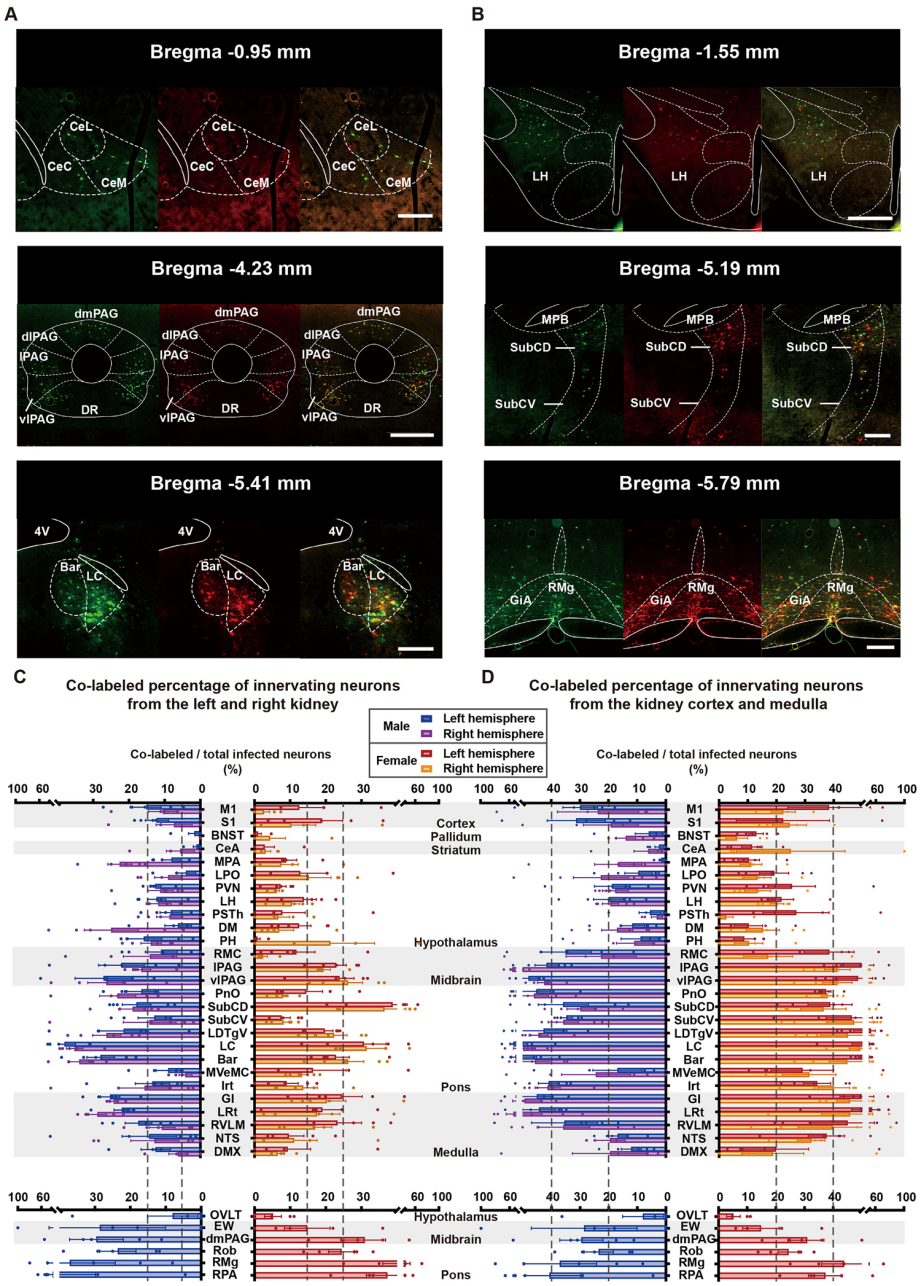
Comparison of overlap percentage of EGFP^+^ and mRuby^+^ neurons. **A** Comparison of the percentage of neurons innervating both the left and right kidney in each brain region and its sex differences. Brain regions were divided into three clusters which contained low (<15%), moderate (15%–25%), and high (>25%) percentages of co-labeled neurons. Panel **C** shows the representative images of each cluster. **B** Comparison of the percentage of neurons innervating both kidney cortex and medulla in each brain region, and its sex differences. Brain regions were divided into three clusters which contained low (<20%), moderate (20%–40%), and high (>40%) percentages of co-labeled neurons. Panel **D** shows the representative images of each cluster. Data in **A** and **B** are shown as the mean ± SEM. Data were analyzed by two-way ANOVA with Šídák’s *post hoc* test. Scale bars, CeA, LC, SubC, and RMg = 250 μm; PAG and LH = 500 μm.

Statistical analysis was performed with the ratio of neurons that simultaneously innervate both left and right kidneys, as well as the cortex and medulla of the left kidney, and across all brain regions (Fig. 8C, D). The brain regions were classified into three clusters based on the ratio of co-labeled neurons to total neurons. For the innervation of the left and right kidneys, the co-labeling rate was defined as low (<15%), moderate (15%–25%), and high (>25%). For the innervation of the renal cortex and medulla, the co-labeling rate was defined as low (<20%), moderate (20%–40%) and high (>40%). The CeA was a region with low co-labeling rates, while the PAG had a moderate co-labeling rate, and the locus coeruleus (LC) had high co-labeling rates (Fig. 8A). Regarding co-labeling neurons underlying renal cortex and medulla connections, the LH had low co-labeling rates, while the dorsal and ventral subcoeruleus nucleus (SubCD and SubCV) had moderate co-labeling rates, and the raphe magnus nucleus (RMg) and gigantocellular reticular nucleus (Gi) had high co-labeling rates (Fig. 8B). However, the results showed no statistical differences in the proportion of bilateral connections or cortex/medulla connections across all brain regions in both male and female mouse brains.

Overall, the proportion of brain regions that co-innervate the left and right kidneys was much lower than those that co-innervate the cortex and medulla, but both displayed similar trends among all brain regions. In both experiments viral tracing of left-right kidney innervation and renal cortex-medulla innervation showed high co-labeling rates in the midbrain, pons, and medulla compared to the forebrain. This suggests that there may be further delicate and complicated neural regulations on renal functions.

## Discussion

In this study, the connectivity between the kidneys and the brain was systematically mapped in both male and female mice, considering both left and right kidneys as well as the renal cortex and medulla. The results showed that certain brain regions, such as the PVN, PSTh, RMC, and M1/S1, displayed a highly unilateral biased pattern. No significant differences were found in the distribution of renal cortex and medulla-connecting neurons in the brain. Mild sex differences were found in kidney-connecting neuron distribution. Additionally, moderate co-innervating rates (<60%) were observed for both kidney and kidney substructures in most brain regions. This study comprehensively described up to 34 brain regions that connect to both kidneys and, for the first time, took into account substructure factors and sex dimorphism that may contribute to kidney innervation.

PRV-labeled neurons in the cortex were mainly found in the M1 and S1 regions, which is similar to what was reported by Levinthal and Strick *et al*. [18,19] for rabies virus-infected neurons related to the stomach and adrenal gland in monkey and rat brains. These researchers suggested that the motor cortex plays a role in predictive feedforward regulation of visceral functions, which is essential for allostatic regulation. Yao *et al*. [20] proposed that the M1 layer V serves as a relay delivering higher cortical decisions to the micturition center to control the voluntary motor of detrusor in the bladder. In this case, the M1 controls the muscular components of the bladder, while other organs like the adrenal gland and kidney lack skeletomuscular structures that directly execute contraction or relaxation. It is still unknown whether there is a visceral representation like a skeletomotor representation. However, classic physiological experiments have reported that activation of the M1 induced a decrease in renal blood flow [21], and the clinical trial of transcranial direct current stimulation on the M1 for stroke rehabilitation also increases sympathetic activity [22].

In addition to the M1/S1, we also found spatial characteristics in three nuclei that have seldom been discussed in previous studies: the RMC, Ew, and PSTh. Among them, only two published papers mentioned the RMC [23,24]. A typical stripped distribution was found in the ventral part of the contralateral side of the RMC, the magnocellular part of the RMC, with high spatial heterogeneity. The RMC displayed a typically contralateral distribution pattern where the right RMC connected to the left kidney and vice versa, which had been reported before [24]. The magnocellular part of the RMC is known to initiate the rubrospinal tract that controls sensorimotor functions. The non-motor functions of the RMC have primarily been discussed in pain research, where they demonstrate antinociceptive effects that occur after the activation of the PAG and nucleus raphe magnus, as well as the lateral reticular nucleus [25]. However, the role of RMC in renal function regulation or autonomic output is still unclear.

The Ew and the PSTh are clearly and densely labeled by PRV, yet they have seldom been discussed in the field of kidney connection. The Ew is located in the midbrain underneath the PAG and is involved in stress responses, modulating cardiovascular functions, energy metabolism thermogenesis, and food intake [26]. It has been reported to connect polysynaptically with adipose tissue [27], spleen [28], adrenal gland [29], and even the kidney [24]. Cano *et al*. [30] demonstrated that the Ew projects sympathetic preganglionic neurons in the spinal cord using anterograde tracer. The Ew is probably embedded within the allostasis model integrating internal milieu and external information by acting as not only a parasympathetic preganglionic nucleus but also a central presympathetic nucleus. However, further investigation is needed to understand how the Ew regulates renal functions to maintain homeostasis. The PSTh has emerged as an integrative hub for sensing and modulating autonomic changes and homeostasis in recent years [31]. PRV retrograde tracing from several visceral organs displayed intense labeling in the PSTh (not shown in the current article), indicating its possible role in orchestrating interoception and autonomic outflow. Although, there is no direct evidence demonstrating that PSTh controls renal functions, activation of it significantly depressed mean arterial pressure, renal sympathetic nerve activity, and heart rate [31]. The role of the Ew and PSTh in visceral organ functions will extend our understanding of interoception and autonomic outflow.

Previous studies have revealed kidney-brain connectivity using tracing dye and viral tools. However, comprehensive mapping has been limited by survival time and virus injection strategy. In previous studies, the PRV virus was injected into the renal parenchyma at random points without distinguishing substructures. Evidence from viral tracing strategies has demonstrated that different populations of ganglion neurons and even spinal neurons innervate different parts of the kidney [32,33]. In this study, we focused on the central connection pattern of the renal cortex and medulla, which have different structures and functions and have rich sympathetic innervation controlling hemodynamics, renin release, and sodium reabsorption [2]. On the other hand, the medulla has less sympathetic innervation but rich sensory nerves in the pelvis wall. Most of the brain regions showed a similar intensity of connection with both the renal cortex and medulla. However, the LH and PSTh displayed a higher connection with the cortex in male brains compared to female brains (Fig. 7B, C). The LH and PSTh are two nuclei that regulate energy metabolism and cardiovascular activities. They have a more delicate division of work in regulating vasculature, tubular, and secretion functions to maintain homeostasis.

It is well-established that there are sex differences in kidney diseases, with men progressing faster than women in chronic kidney diseases and hypertension being more common in young men than in age-matched women [14,34]. These differences are largely attributed to sex hormones such as estrogen, androgen, and progesterone. Post-menopause females, both human and animal models, have been shown to have blood pressure levels similar to males and a higher risk for hypertension than pre-menopause females [35]. Additionally, there are differences in renal physiology between males and females. For example, the size of the renal cortex and proximal tubules is larger in males, while the renal medulla is larger in females as reported in Wistar-Tokyo rats [36]. However, neural activity also varies between males and females and may contribute to the sex differences in renal and cardiovascular function. Pre-menopause women have been shown to have lower sympathetic activity than men [37], and even in an ischemia/reperfusion injury mouse model, sympathetic activity declines faster in young female mice [38]. The PVN and RVLM, which are major sources of excitatory drive to sympathetic outflow, displayed fewer PRV-labeled neurons in female mice than in males, although this may be due to the non-cell type-specific strategy used in the current study. We also found a hemisphere-biased distribution of left kidney-connected neuron numbers in the female LH but not in males. The LH is the central controller of energy metabolism and sympathetic activity. The degree to which the nervous system contributes to sexual dimorphism in renal and cardiovascular functions still lacks evidence. However, our study adds to possible mechanisms underlying sex differences in renal functions beyond sex hormones.

Our current study still has some limitations. First, we did not observe infected neurons in the subfornical organ (SFO) as reported by a previous study [39] that used a combinatorial monosynaptic virus strategy to trace the renal afferent pathway from the kidney to T9–11 spinal dorsal horn to the SFO. In our tracing samples, we didn’t observe labeled neurons in the dorsal horn but dense infection mainly in the intermediolateral column and lamina 10 at T11–L1 sections. This is probably because the PRV-Bartha strain mainly propagates only along efferent nerves, as was reported before [40]. Secondly, we observed individual significances in both the PRV-EGFP and PRV-mRuby despite sealing the needle with kwit-sil using a constant injection velocity and depth from the kidney surface. We explain this as being due to different densities of nerve terminals around the injection site.

In conclusion, our study provided a comprehensive atlas of kidney-brain connectivity, taking into account different sides of the kidney, different substructures of the kidney, and different sexes. This will help better understand how the brain orchestrates different organs to maintain homeostasis and benefit the development of novel intervention strategies for mental and peripheral comorbidities.

## Supplementary Information

Below is the link to the electronic supplementary material.

Supplementary file1 (PDF 699 KB)

## References

[1] Carlström M, Wilcox CS, Arendshorst WJ. Renal autoregulation in health and disease. Physiol Rev 2015, 95: 405–511. 25834230 10.1152/physrev.00042.2012 PMC4551215

[2] DiBona GF, Kopp UC. Neural control of renal function. Physiol Rev 1997, 77: 75–197. 9016301 10.1152/physrev.1997.77.1.75

[3] Hausberg M, Kosch M, Harmelink P, Barenbrock M, Hohage H, Kisters K. Sympathetic nerve activity in end-stage renal disease. Circulation 2002, 106: 1974–1979. 12370222 10.1161/01.cir.0000034043.16664.96

[4] Minatoguchi S. Heart failure and its treatment from the perspective of sympathetic nerve activity. J Cardiol 2022, 79: 691–697. 34924233 10.1016/j.jjcc.2021.11.016

[5] Sata Y, Head GA, Denton K, May CN, Schlaich MP. Role of the sympathetic nervous system and its modulation in renal hypertension. Front Med 2018, 5: 82. 10.3389/fmed.2018.00082 PMC5884873 29651418

[6] Thorp AA, Schlaich MP. Relevance of sympathetic nervous system activation in obesity and metabolic syndrome. J Diabetes Res 2015, 2015: 341583. 26064978 10.1155/2015/341583 PMC4430650

[7] Barbato E, Azizi M, Schmieder RE, Lauder L, Böhm M, Brouwers S, *et al*. Renal denervation in the management of hypertension in adults. A clinical consensus statement of the ESC Council on Hypertension and the European Association of Percutaneous Cardiovascular Interventions (EAPCI). Eur Heart J 2023, 44: 1313–1330. 10.1093/eurheartj/ehad054 36790101

[8] Schramm LP, Strack AM, Platt KB, Loewy AD. Peripheral and central pathways regulating the kidney: A study using pseudorabies virus. Brain Res 1993, 616: 251–262. 7689411 10.1016/0006-8993(93)90216-a

[9] Gattone VH 2nd, Marfurt CF, Dallie S. Extrinsic innervation of the rat kidney: A retrograde tracing study. Am J Physiol 1986, 250: F189–F196. 3753828 10.1152/ajprenal.1986.250.2.F189

[10] Huang J, Weiss ML. Characterization of the central cell groups regulating the kidney in the rat. Brain Res 1999, 845: 77–91. 10529446 10.1016/s0006-8993(99)01937-x

[11] Stachenfeld NS, Splenser AE, Calzone WL, Taylor MP, Keefe DL. Sex differences in osmotic regulation of AVP and renal sodium handling. J Appl Physiol 2001, 91: 1893–1901. 11568177 10.1152/jappl.2001.91.4.1893

[12] Hu R, McDonough AA, Layton AT. Sex differences in solute transport along the nephrons: Effects of Na^+^ transport inhibition. Am J Physiol Renal Physiol 2020, 319: F487–F505. 32744084 10.1152/ajprenal.00240.2020 PMC7509281

[13] Tanaka R, Tsutsui H, Ohkita M, Takaoka M, Yukimura T, Matsumura Y. Sex differences in ischemia/reperfusion-induced acute kidney injury are dependent on the renal sympathetic nervous system. Eur J Pharmacol 2013, 714: 397–404. 23872383 10.1016/j.ejphar.2013.07.008

[14] Bairey Merz CN, Dember LM, Ingelfinger JR, Vinson A, Neugarten J, Sandberg KL, *et al*. Sex and the kidneys: Current understanding and research opportunities. Nat Rev Nephrol 2019, 15: 776–783. 31586165 10.1038/s41581-019-0208-6 PMC7745509

[15] Jia F, Lv P, Miao H, Shi X, Mei H, Li L, *et al*. Optimization of the fluorescent protein expression level based on pseudorabies virus Bartha strain for neural circuit tracing. Front Neuroanat 2019, 13: 63. 31281245 10.3389/fnana.2019.00063 PMC6597954

[16] Kucera M, Wistrela E, Pfusterschmied G, Ruiz-Díez V, Manzaneque T, Luis Sánchez-Rojas J, *et al*. Characterization of a roof tile-shaped out-of-plane vibrational mode in aluminum-nitride-actuated self-sensing micro-resonators for liquid monitoring purposes. Appl Phys Lett 2014, 104: 233501.

[17] Hahn JD, Gao L, Boesen T, Gou L, Hintiryan H, Dong HW. Macroscale connections of the mouse lateral preoptic area and anterior lateral hypothalamic area. J Comp Neurol 2022, 530: 2254–2285. 35579973 10.1002/cne.25331 PMC9283274

[18] Levinthal DJ, Strick PL. The motor cortex communicates with the kidney. J Neurosci 2012, 32: 6726–6731. 22573695 10.1523/JNEUROSCI.0406-12.2012 PMC3363289

[19] Levinthal DJ, Strick PL. Multiple areas of the cerebral cortex influence the stomach. Proc Natl Acad Sci U S A 2020, 117: 13078–13083. 32434910 10.1073/pnas.2002737117 PMC7293610

[20] Yao J, Zhang Q, Liao X, Li Q, Liang S, Li X, *et al*. A corticopontine circuit for initiation of urination. Nat Neurosci 2018, 21: 1541–1550. 30361547 10.1038/s41593-018-0256-4

[21] Wall PD, Pribram KH. Trigeminal neurotomy and blood pressure responses from stimulation of lateral cerebral cortex of *Macaca mulatta*. J Neurophysiol 1950, 13: 409–412. 14784864 10.1152/jn.1950.13.6.409

[22] Clancy JA, Johnson R, Raw R, Deuchars SA, Deuchars J. Anodal transcranial direct current stimulation (tDCS) over the motor cortex increases sympathetic nerve activity. Brain Stimul 2014, 7: 97–104. 24080439 10.1016/j.brs.2013.08.005

[23] Mohanta SK, Peng L, Li Y, Lu S, Sun T, Carnevale L, *et al*. Neuroimmune cardiovascular interfaces control atherosclerosis. Nature 2022, 605: 152–159. 35477759 10.1038/s41586-022-04673-6

[24] Cano G, Card JP, Sved AF. Dual viral transneuronal tracing of central autonomic circuits involved in the innervation of the two kidneys in rat. J Comp Neurol 2004, 471: 462–481. 15022264 10.1002/cne.20040

[25] Basile GA, Quartu M, Bertino S, Serra MP, Boi M, Bramanti A, *et al*. Red nucleus structure and function: From anatomy to clinical neurosciences. Brain Struct Funct 2021, 226: 69–91. 33180142 10.1007/s00429-020-02171-x PMC7817566

[26] Cano G, Hernan SL, Sved AF. Centrally projecting edinger-westphal nucleus in the control of sympathetic outflow and energy homeostasis. Brain Sci 2021, 11: 1005. 34439626 10.3390/brainsci11081005 PMC8392615

[27] Zhang Y, Kerman IA, Laque A, Nguyen P, Faouzi M, Louis GW, *et al*. Leptin-receptor-expressing neurons in the dorsomedial hypothalamus and Median preoptic area regulate sympathetic brown adipose tissue circuits. J Neurosci 2011, 31: 1873–1884. 21289197 10.1523/JNEUROSCI.3223-10.2011 PMC3069639

[28] Cano G, Sved AF, Rinaman L, Rabin BS, Card JP. Characterization of the central nervous system innervation of the rat spleen using viral transneuronal tracing. J Comp Neurol 2001, 439: 1–18. 11579378 10.1002/cne.1331

[29] Kerman IA, Akil H, Watson SJ. Rostral elements of sympatho-motor circuitry: A virally mediated transsynaptic tracing study. J Neurosci 2006, 26: 3423–3433. 16571749 10.1523/JNEUROSCI.5283-05.2006 PMC6673864

[30] Dos Santos Júnior ED, Da Silva AV, Da Silva KRT, Haemmerle CAS, Batagello DS, Da Silva JM, *et al*. The centrally projecting Edinger-Westphal nucleus—I: Efferents in the rat brain. J Chem Neuroanat 2015, 68: 22–38. 26206178 10.1016/j.jchemneu.2015.07.002

[31] Shah T, Dunning JL, Contet C. At the heart of the interoception network: Influence of the parasubthalamic nucleus on autonomic functions and motivated behaviors. Neuropharmacology 2022, 204: 108906. 34856204 10.1016/j.neuropharm.2021.108906 PMC8688299

[32] Maeda S, Fujihira M, Minato Y, Kuwahara-Otani S, Tanaka K, Hayakawa T, *et al*. Differential distribution of renal nerves in the sympathetic Ganglia of the rat. Anat Rec 2017, 300: 2263–2272. 10.1002/ar.23680 28834374

[33] Huang J, Chowhdury SI, Weiss ML. Distribution of sympathetic preganglionic neurons innervating the kidney in the rat: PRV transneuronal tracing and serial reconstruction. Auton Neurosci 2002, 95: 57–70. 11871786 10.1016/s1566-0702(01)00356-3

[34] Drury ER, Wu J, Gigliotti JC, Le TH. Sex differences in blood pressure regulation and hypertension: Renal, hemodynamic, and hormonal mechanisms. Physiol Rev 2024, 104: 199–251. 37477622 10.1152/physrev.00041.2022 PMC11281816

[35] Ji H, Kim A, Ebinger JE, Niiranen TJ, Claggett BL, Bairey Merz CN, *et al*. Sex differences in blood pressure trajectories over the life course. JAMA Cardiol 2020, 5: 19–26. 31940010 10.1001/jamacardio.2019.5306 PMC6990675

[36] Oudar O, Elger M, Bankir L, Ganten D, Ganten U, Kriz W. Differences in rat kidney morphology between males, females and testosterone-treated females. Ren Physiol Biochem 1991, 14: 92–102. 1707550 10.1159/000173392

[37] Jarvis SS, VanGundy TB, Galbreath MM, Shibata S, Okazaki K, Reelick MF, *et al*. Sex differences in the modulation of vasomotor sympathetic outflow during static handgrip exercise in healthy young humans. Am J Physiol Regul Integr Comp Physiol 2011, 301: R193–R200. 21508291 10.1152/ajpregu.00562.2010 PMC3129874

[38] Hosszu A, Fekete A, Szabo AJ. Sex differences in renal ischemia-reperfusion injury. Am J Physiol Renal Physiol 2020, 319: F149–F154. 32567347 10.1152/ajprenal.00099.2020

[39] Cao W, Yang Z, Liu X, Ren S, Su H, Yang B, *et al*. A kidney-brain neural circuit drives progressive kidney damage and heart failure. Signal Transduct Target Ther 2023, 8: 184. 37169751 10.1038/s41392-023-01402-x PMC10175540

[40] Brittle EE, Reynolds AE, Enquist LW. Two modes of pseudorabies virus neuroinvasion and lethality in mice. J Virol 2004, 78: 12951–12963. 15542647 10.1128/JVI.78.23.12951-12963.2004 PMC525033

