# Supplementary material for: Sex-Dimorphic Kidney-Brain Connectivity Map of Mice
Source: Neurosci Bull. 2024 Jun 19;40(10):1445–57. doi: 10.1007/s12264-024-01240-z (PMC11422536; doi:10.1007/s12264-024-01240-z)
Supplement: Supplementary file 1 — Supplementary file1 (PDF 699 KB) [file 12264_2024_1240_MOESM1_ESM.pdf]

Supplementary Materials

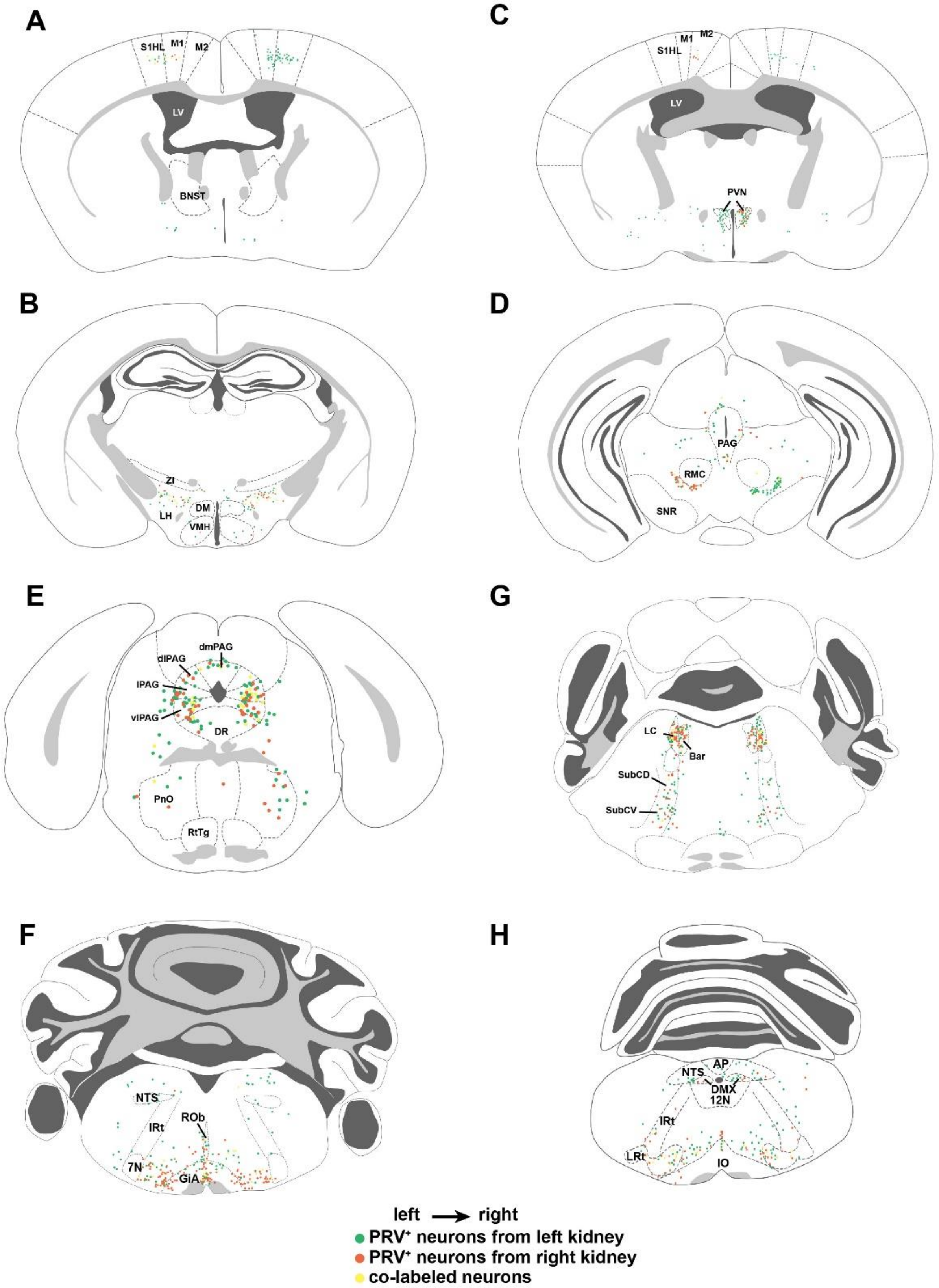

Fig. S1 Schematic diagram of the left and right kidney-related neuron distribution in the mouse brain.

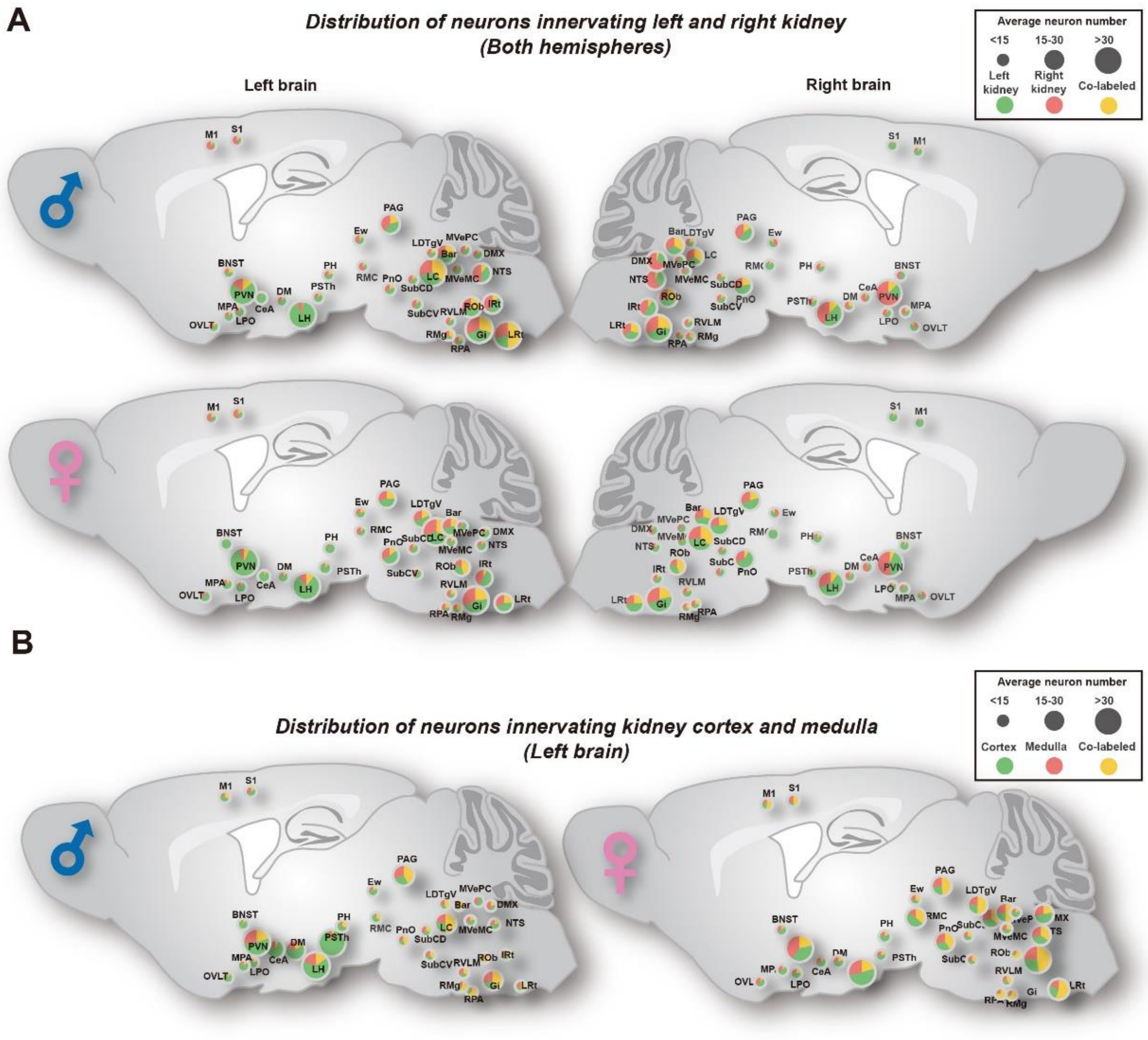

Fig. S2 Distribution of neurons innervating left and right kidneys, or the kidney cortex and medulla in male and female C57BL/6J mice. A, Distribution of neurons connecting to the left and right kidney. B, Distribution of neurons connecting to the kidney cortex and medulla.

Supplementary Tables

Table S1 Number of PRV-labeled neurons innervating the left or right kidney in each brain region. EGFP labels neurons innervating the left kidney; mRuby labels the neurons innervating the right kidney (*n* = 5). Data are shown as the mean ± SEM.

	Male						Female					
	Left brain			Right brain			Left brain			Right brain		
	EGFP	mRuby	co/total %	EGFP	mRuby	co/total %	EGFP	mRuby	co/total %	EGFP	mRuby	co/total %
**M1**	3.483 ±1.908	9.267 ±6.784	15.058 ±8.931	6.417 ±5.688	2.300 ±2.405	10.508 ±9.188	2.967 ±2.799	4.083 ±4.074	12.480 ±15.149	11.717 ±8.053	1.783 ±2.582	2.587 ±3.601
**S1**	2.500 ±2.672	5.417 ±3.985	12.192 ±7.235	5.967 ±6.573	0.533 ±0.767	7.374 ±11.347	4.683 ±1.649	6.883 ±4.655	18.816 ±13.235	10.617 ±5.546	1.350 ±1.137	10.301 ±15.405
**BNST**	0.200 ±0.447	0.133 ±0.298	1.667 ±3.727	0.067 ±0.149	0.067 ±0.149	0	6.200 ±3.877	0.200 ±0.298	0.939 ±2.101	3.733 ±3.897	0.867 ±1.325	4.340 ±9.704
**CeA**	9.867 ±14.017	0.850 ±0.914	1.078 ±2.077	1.317 ±1.449	2.100 ±1.698	5.635 ±8.723	4.383 ±5.413	0.500 ±0.984	2.788 ±6.234	4.867 ±4.730	8.233 ±16.805	3.017 ±3.348
**MPA**	5.000 ±1.612	2.017 ±1.677	8.060 ±6.120	3.167 ±1.969	2.867 ±2.488	22.550 ±7.565	6.35 ±3.060	2.083 ±1.678	8.804 ±6.823	4.050 ±3.028	1.867 ±1.505	7.438 ±10.767
**LPO**	2.533 ±1.758	1.267 ±1.256	4.000 ±5.963	1.600 ±1.011	1.543 ±0.615	9.000 ±8.608	7.050 ±4.327	1.683 ±1.890	12.761 ±16.836	6.117 ±3.272	3.583 ±3.194	15.193 ±14.046
**PVN**	38.733 ±10.267	13.417 ±7.708	12.599 ±3.434	9.967 ±4.534	26.583 ±1.294	11.167 ±7.955	39.367 ±14.660	5.800 ±3.529	7.241 ±3.658	14.717 ±17.938	16.967 ±5.554	6.174 ±1.773
**LH**	23.000 ±5.927	11.133 ±7.368	12.236 ±5.728	17.183 ±5.756	16.067 ±11.388	11.734 ±7.732	42.900 ±25.781	9.767 ±4.928	13.774 ±7.982	24.823 ±19.081	13.530 ±7.447	10.418 ±5.074
**PSTh**	4.867 ±3.783	2.167 ±1.167	8.231 ±6.874	2.367 ±2.056	2.317 ±2.135	8.613 ±8.172	6.150 ±4.950	1.317 ±2.131	7.742 ±14.477	2.967 ±2.174	1.333 ±1.929	6.611 ±7.202
**DM**	5.000 ±3.758	2.300 ±2.110	6.241 ±8.295	4.033 ±2.103	4.467 ±3.324	24.952 ±16.891	12.067 ±6.153	2.867 ±3.079	12.436 ±7.595	6.400 ±1.935	5.600 ±5.150	6.872 ±9.291
**PH**	6.700 ±3.429	4.617 ±3.360	15.794 ±11.162	5.283 ±3.392	4.533 ±3.526	13.829 ±6.343	4.917 ±3.997	0.600 ±0.548	0.784 ±1.754	3.333 ±2.739	1.400 ±2.047	21.222 ±27.923

**RMC**	4.050 ±3.812	8.133 ±4.873	10.990 ±9.923	11.617 ±6.350	1.800 ±1.121	14.108 ±16.713	6.950 ±5.286	6.700 ±3.840	11.702 ±11.796	19.067 ±10.882	1.483 ±1.100	2.371 ±2.770
**lPAG**	11.067 ±2.919	10.800 ±2.968	22.108 ±14.319	10.800 ±3.849	10.200 ±4.741	16.469 ±5.654	19.200 ±7.988	14.667 ±7.750	22.850 ±6.407	19.867 ±9.350	15.000 ±8.216	19.310 ±4.424
**vlPAG**	12.933 ±1.754	12.667 ±3.333	27.083 ±17.933	12.067 ±2.454	11.667 ±4.123	26.268 ±11.844	16.000 ±5.831	11.600 ±4.092	23.768 ±9.307	15.400 ±6.614	12.267 ±5.499	26.160 ±9.290
**PnO**	9.133 ±3.602	6.933 ±3.427	16.479 ±10.608	12.933 ±10.186	10.400 ±6.970	23.162 ±11.309	17.267 ±7.002	12.667 ±4.690	14.464 ±10.376	15.867 ±5.490	9.267 ±3.370	7.880 ±3.384
**SubCD**	7.867 ±2.231	7.200 ±0.691	17.876 ±8.929	8.933 ±1.553	7.400 ±2.681	18.928 ±7.845	8.333 ±5.442	5.533 ±2.399	13.426 ±8.406	10.200 ±8.204	6.533 ±3.540	12.629 ±10.051
**SubCV**	6.600 ±2.074	5.800 ±2.142	12.883 ±10.197	6.800 ±2.063	6.000 ±2.198	14.233 ±16.510	8.667 ±6.625	6.600 ±4.639	7.537 ±5.553	8.933 ±7.436	6.800 ±3.159	8.103 ±3.854
**LDTgV**	6.667 ±0.913	5.600 ±1.256	21.456±15.343	7.333 ±1.986	7.267 ±2.350	26.248 ±8.773	14.200 ±5.984	10.267 ±2.862	19.644 ±5.222	13.067 ±6.269	8.733 ±2.862	22.291 ±8.195
**LC**	25.200 ±3.768	23.000 ±1.616	38.024 ±6.834	19.000 ±6.566	19.067 ±8.716	35.199 ±5.057	25.700 ±12.427	22.333 ±8.003	30.638 ±14.057	23.100 ±14.322	19.433 ±7.566	31.388 ±12.176
**Bar**	12.600 ±2.278	9.800 ±2.479	27.940 ±8.564	11.667 ±2.549	11.400 ±3.004	33.853 ±7.279	14.800 ±6.529	11.133 ±4.112	22.749 ±8.637	12.867 ±9.239	10.000 ±4.619	26.247 ±9.924
**MVeMC**	9.400 ±5.565	6.067 ±3.539	8.977 ±7.745	8.200 ±4.897	6.200 ±2.864	4.171 ±4.038	8.333 ±5.977	3.267 ±2.060	16.316 ±20.992	7.267 ±5.713	3.600 ±3.227	13.250 ±4.150
**Irt**	10.867 ±4.350	9.933 ±3.040	13.432 ±10.757	11.267 ±5.107	9.733 ±3.353	15.582 ±16.394	10.400 ±5.570	8.800 ±3.962	8.963 ±7.319	9.167 ±7.506	6.533 ±3.548	13.602 ±9.064
**GI**	32.933 ±10.943	29.267 ±6.689	25.270 ±6.027	32.467 ±13.010	27.667 ±9.542	24.237 ±2.165	25.467 ±10.798	21.467 ±8.780	24.465 ±12.881	23.867 ±13.031	18.267 ±7.436	20.248 ±12.081
**LRt**	26.600 ±34.848	15.333 ±2.248	21.971 ±4.973	14.333 ±3.342	13.200 ±3.509	28.790 ±5.543	17.400 ±14.880	13.800 ±11.789	18.957 ±12.011	18.133 ±16.723	13.200 ±11.890	17.465 ±14.546
**RVLM**	5.333 ±2.095	4.667 ±1.374	17.341 ±8.341	5.400 ±1.801	5.133 ±1.464	10.721 ±8.105	2.600 ±1.673	2.733 ±2.074	23.204 ±15.255	6.333 ±7.196	4.400 ±5.464	16.959 ±10.578
**NTS**	10.800 ±5.321	13.400 ±14.734	14.298 ±17.071	9.600 ±4.850	16.333 ±17.203	12.789 ±15.033	10.867 ±5.475	5.333 ±4.333	9.585 ±9.549	8.833 ±6.137	5.417 ±3.047	11.060 ±14.519

**DMX**	8.000 ±0.943	5.267 ±3.811	12.518 ±9.126	4.467 ±1.726	10.867 ±4.363	6.272 ±5.778	9.667 ±5.715	4.200 ±2.912	9.251 ±14.570	6.667 ±5.132	7.533 ±3.211	6.429 ±4.386

	**Male**			**Female**		
	**EGFP**	**mRuby**	**co/total %**	**EGFP**	**mRuby**	**co/total %**
**OVLT**	2.467 ±2.268	1.767 ±1.526	7.017 ±14.729	6.700 ±5.239	2.667 ±3.089	4.667 ±5.224
**Ew**	1.867 ±0.701	1.633 ±0.711	27.963 ±40.967	2.500 ±1.576	1.750 ±1.601	12.614 ±14.610
**dmPAG**	7.267 ±5.013	7.600 ±5.372	32.554 ±18.347	10.000 ±5.142	7.800 ±4.093	31.838 ±14.009
**ROb**	10.883 ±1.040	10.800 ±1.574	23.796 ±12.167	10.000 ±7.219	10.400 ±6.304	25.054 ±8.371
**RPA**	6.533 ±1.850	7.133 ±1.574	32.594 ±23.050	6.267 ±2.477	6.000 ±1.795	38.946 ±13.708
**RMg**	9.467 ±3.429	8.867 ±4.233	34.116 ±30.242	8.067 ±6.970	6.467 ±3.288	45.864 ±15.746

M1, Primary motor cortex; S1, Primary somatosensory cortex; OVLT, Organum vasculosum laminae terminalis; BNST, Bed nucleus of the stria terminalis; CeA, Central nucleus of the amygdala; MPA, Medial preoptic area; LPO, Lateral preoptic area; PVN, Paraventricular nucleus; LH, Lateral hypothalamic area; PSTh, Parasubthalamic nucleus; DM, Dorsomedial hypothalamic; PH, Posterior hypothalamic nucleus; RMC, Red nucleus, magnocellular part; EW, Edinger-Westphal nucleus; dmPAG, Dorsomedial periaqueductal gray; lPAG, Lateral periaqueductal gray; vlPAG, Ventrolateral periaqueductal gray; PnO, Oral pontine reticular nucleus; SubCD, Dorsal subcoeruleus nucleus; SubCV, Ventral subcoeruleus nucleus; LDTgV, Laterodorsal tegmental nucleus, ventral part; ROb, Raphe obscurus nucleus; RMg, Raphe magnus nucleus; RPA, Raphe pallidus nucleus; LC, Locus coeruleus; Bar, Barrington’s nucleus; MVePC, Medial vestibular nucleus, parvicellular part; MVeMC, Medial vestibular nucleus, magnocellular part; IRt, Intermediate reticular nucleus; Gi, Gigantocellular reticular nucleus; LRt, Lateral reticular nucleus; RVLM, Rostral ventrolateral medulla; NTS, Solitary nucleus; DMV, Dorsal motor nucleus of the vagus.

Table S2 Number of PRV-labeled neurons innervating the kidney cortex or medulla in each brain region. EGFP labels neurons innervating the kidney cortex; mRuby labels the neurons innervating the kidney medulla (*n* = 5). Data are shown as the mean ± SEM.

	**Male**						**Female**					
	**Left brain**			**Right brain**			**Left brain**			**Right brain**		
	**EGFP**	**mRuby**	**co/total %**	**EGFP**	**mRuby**	**co/total %**	**EGFP**	**mRuby**	**co/total %**	**EGFP**	**mRuby**	**co/total %**
**M1**	6.267 ±8.005	3.700 ±5.207	23.691 ±31.677	12.133 ±14.983	8.933 ±13.580	29.892 ±13.545	4.000 ±3.559	3.400 ±3.022	38.241 ±28.640	19.200 ±15.830	11.773 ±7.986	22.333 ±8.429
**S1**	4.933 ±8.496	3.600 ±6.954	19.222 ±21.479	9.467 ±7.999	5.467 ±3.863	31.248 ±22.678	5.667 ±9.557	7.533 ±10.558	22.267 ±32.487	28.147 ±16.511	23.387 ±31.443	24.541 ±11.873
**BNST**	8.917 ±7.929	2.467 ±1.579	13.927 ±7.365	11.683 ±12.609	0.717 ±0.960	5.902 ±10.762	6.633 ±4.519	4.233 ±4.870	12.913 ±5.676	2.200 ±1.850	1.300 ±1.445	6.190 ±8.518
**CeA**	14.873 ±14.200	2.947 ±2.395	6.100 ±11.259	32.533 ±36.777	1.147 ±1.440	2.373 ±3.260	7.267 ±7.733	3.983 ±5.899	11.424 ±7.959	2.617 ±1.092	2.383 ±1.965	24.942 ±42.340
**MPA**	6.250 ±5.051	1.983 ±2.261	16.578 ±18.507	8.283 ±11.783	0.750 ±0.920	1.586 ±1.729	5.460 ±1.190	2.850 ±2.927	10.323 ±8.467	3.230 ±2.947	2.360 ±3.473	11.144 ±8.471
**LPO**	4.150 ±1.278	2.100 ±1.140	22.631 ±21.609	5.250 ±5.499	1.033 ±0.749	9.560 ±10.783	6.390 ±2.449	3.800 ±4.578	19.199 ±11.382	3.580 ±1.825	2.330 ±2.608	13.380 ±10.615
**PVN**	36.333 ±24.045	23.900 ±11.571	17.621 ±10.054	23.817 ±22.434	10.233 ±6.777	18.738 ±13.858	29.900 ±11.708	30.417 ±16.102	25.340 ±18.504	17.567 ±4.753	20.100 ±14.763	13.423 ±10.264
**LH**	41.200 ±21.725	19.333 ±8.456	19.336 ±6.369	37.067 ±29.734	13.467 ±7.373	19.726 ±12.354	30.983 ±12.351	20.633 ±15.753	21.686 ±9.697	24.583 ±8.313	17.400 ±14.348	20.228 ±5.366
**PSTh**	24.833 ±29.348	1.767 ±1.588	3.134 ±4.306	27.800 ±32.856	2.133 ±2.090	5.511 ±8.566	6.000 ±5.977	3.633 ±5.521	26.842 ±25.825	1.733 ±2.100	1.267 ±2.650	2.450 ±5.478
**DM**	8.467 ±9.332	4.000 ±2.248	16.979 ±12.172	9.333 ±9.107	2.333 ±1.354	11.703 ±9.538	8.733 ±6.946	5.800 ±8.513	10.251 ±11.012	5.983 ±4.327	5.000 ±5.691	15.228 ±12.967
**PH**	6.467 ±7.614	3.267 ±4.106	10.969 ±14.127	4.400 ±3.647	1.267 ±1.640	8.773 ±15.352	6.267 ±4.142	2.533 ±3.286	8.718 ±8.055	4.117 ±4.722	2.283 ±3.011	10.341 ±11.079

	4.633	1.467	22.569	19.717	9.233	34.950	4.400	6.117	38.368	24.167	14.600	17.063
**RMC**	±3.554	±1.516	±27.444	±15.675	±6.094	±19.912	±1.949	±7.118	±19.477	±6.835	±10.409	±19.068
	10.400	9.800	55.398	10.667	10.133	41.577	20.800	21.333	49.661	21.400	18.933	41.297
**lPAG**	±6.759	±6.935	±7.734	±8.410	±6.594	±9.196	±10.219	±14.252	±16.618	±8.355	±12.919	±9.897
	12.333	12.867	42.374	11.800	13.067	47.862	17.733	17.867	48.410	16.667	15.600	41.216
**vlPAG**	±6.737	±7.523	±5.463	±7.198	±7.073	±7.227	±7.949	±9.512	±16.152	±5.578	±8.163	±11.533
	7.467	6.600	45.922	8.667	7.867	45.109	15.067	14.267	37.248	12.467	11.200	37.779
**PnO**	±3.347	±2.929	±11.033	±1.915	±0.767	±11.127	±6.647	±8.597	±3.960	±5.146	±6.951	±4.964
**SubC**	5.333	5.133	29.793	5.133	4.867	35.799	11.133	9.800	38.701	10.000	8.867	36.394
**D**	±2.041	±2.181	±10.600	±2.411	±2.340	±19.561	±5.070	±5.776	±17.212	±5.307	±5.059	±17.733
**SubC**	4.733	4.600	35.739	5.533	5.467	34.654	8.333	8.267	46.194	8.467	6.467	47.029
**V**	±2.842	±2.910	±11.894	±2.588	±3.776	±4.373	±3.520	±5.052	±19.825	±4.628	±3.564	±21.251
**LDTg**	7.733	7.733	44.399	6.933	6.800	42.610	11.000	9.533	52.970	11.733	9.800	44.874
**V**	±2.881	±4.518	±7.351	±2.290	±2.864	±18.054	±4.275	±2.815	±13.428	±4.663	±3.556	±20.977
	18.267	16.800	49.712	15.933	15.133	53.539	22.267	21.967	50.587	20.367	20.767	49.122
**LC**	±7.084	±7.073	±9.402	±7.697	±6.640	±11.920	±9.224	±11.918	±9.371	±9.396	±11.142	±8.258
	8.933	8.133	45.615	9.200	8.667	50.844	13.733	13.533	50.170	13.700	12.600	44.664
**Bar**	±5.575	±3.856	±10.824	±5.460	±2.614	±12.807	±6.387	±8.611	±3.743	±7.725	±8.391	±13.990
**MVeM**	4.667	3.333	24.352	4.000	4.200	16.854	6.133	5.467	29.049	6.267	5.600	31.405
**C**	±1.841	±1.667	±26.947	±1.333	±1.095	±19.248	±1.742	±1.677	±24.360	±2.712	±1.906	±21.039
	6.800	6.133	41.238	6.667	7.133	40.874	11.733	11.133	34.151	10.400	10.333	39.972
**Irt**	±2.873	±2.765	±9.814	±2.991	±2.912	±8.137	±8.084	±8.607	±10.601	±8.295	±9.171	±12.519
	14.733	16.533	49.266	14.467	15.6	44.953	24.333	23.800	49.623	22.267	23.400	45.586
**GI**	±3.585	±4.350	±12.991	±6.384	±6.444	±12.383	±8.663	±6.212	±16.674	±8.105	±4.166	±9.959
	10.200	11.333	53.743	9.600	10.533	44.238	19.333	17.333	54.506	17.800	17.067	45.641
**LRt**	±3.469	±4.989	±23.467	±2.881	±3.564	±12.853	±6.860	±8.663	±18.495	±6.577	±7.131	±22.214
**RVL**	1.933	2.067	35.746	2.600	2.600	35.349	3.333	3.133	44.775	3.667	3.800	39.618
**M**	±1.188	±1.090	±20.499	±1.690	±0.548	±25.769	±0.667	±1.773	±29.784	±1.546	±2.834	±26.891
	8.267	4.800	19.491	10.333	4.867	16.803	12.867	11.333	37.441	12.200	10.200	32.106
**NTS**	±7.057	±3.587	±11.709	±9.715	±3.934	±7.185	±5.108	±4.643	±14.231	±4.482	±4.167	±10.766

**DMX**	6.000 ±6.595	8.133 ±6.488	19.415 ±29.501	7.867 ±8.302	7.333 ±9.664	12.057 ±7.839	14.600 ±9.014	10.533 ±12.294	19.780 ±25.554	15.400 ±12.001	9.867 ±11.843	18.692 ±24.212

	**Male**			**Female**		
	**EGFP**	**mRuby**	**co/total %**	**EGFP**	**mRuby**	**co/total %**
**OVLT**	6.100 ±6.784	1.433 ±1.535	8.370 ±14.219	4.800 ±3.701	3.800 ±3.213	5.536 ±5.279
**Ew dmPAG**	2.267 ±1.765	0.997 ±0.796	9.167 ±12.638	3.267 ±2.465	3.033 ±2.735	40.629 ±25.409
	5.200 ±2.610	4.800 ±2.523	60.370 ±13.926	11.333 ±5.826	10.867 ±7.418	49.038 ±12.332
**ROb**	9.267 ±4.030	8.333 ±3.979	65.773 ±6.897	12.733 ±2.957	14.733 ±4.518	74.508 ±8.432
**RMg**	4.267 ±1.011	4.200 ±1.121	65.221 ±14.340	7.333 ±2.380	7.200 ±1.609	70.200 ±20.973
**RPA**	3.667 ±1.434	3.933 ±1.234	55.667 ±14.701	5.333 ±0.667	6.133 ±1.677	67.824 ±18.855
